# Supplementary figures and images for: Combining OpenStreetMap mapping and route optimization algorithms to inform the delivery of community health interventions at the last mile
Source: PLOS Digit Health. 2024 Nov 7;3(11):e0000621. doi: 10.1371/journal.pdig.0000621 (PMC11542841; doi:10.1371/journal.pdig.0000621)

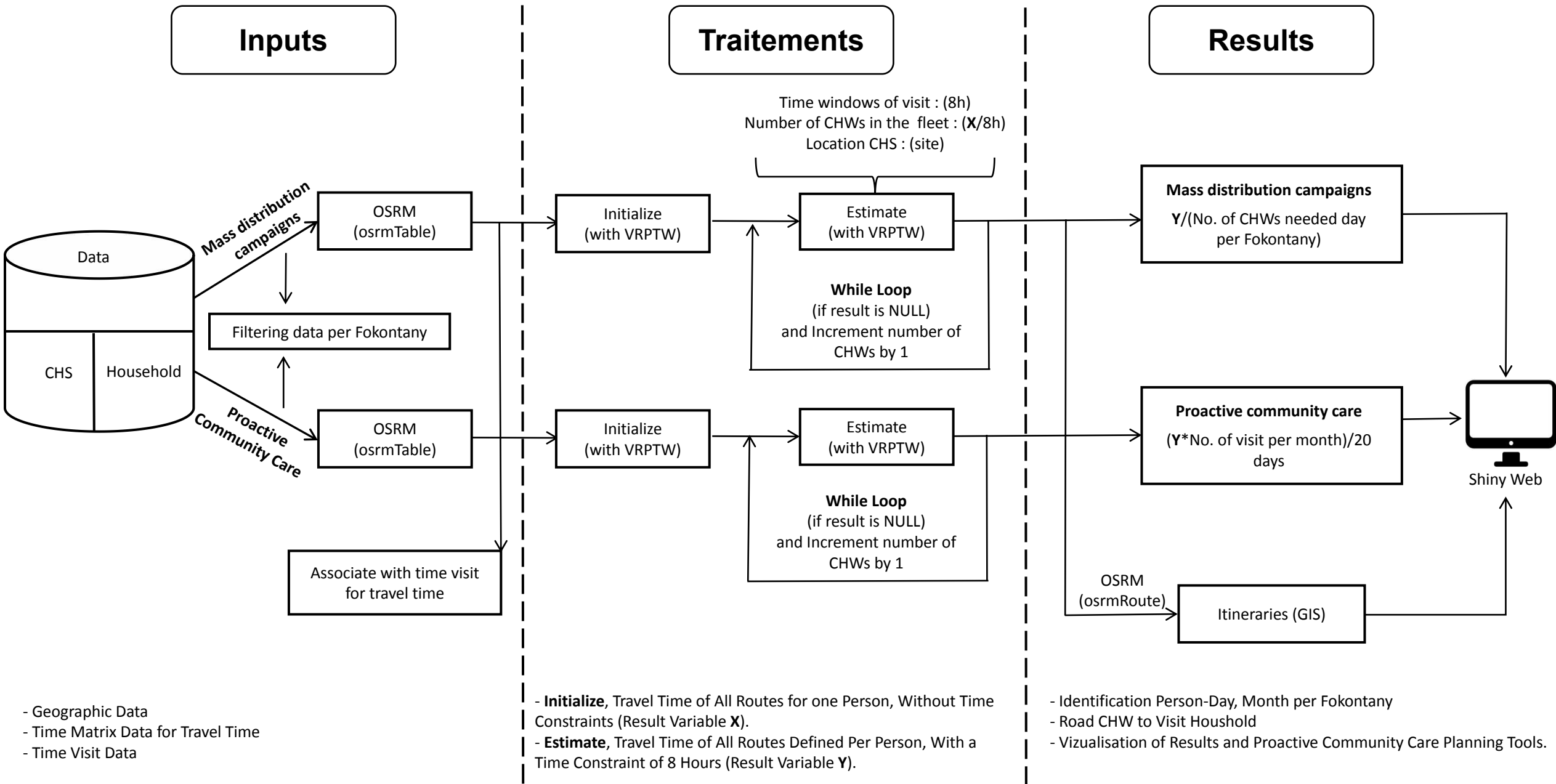

Supplement: S1 Fig — (PDF) [file pdig.0000621.s001.pdf]

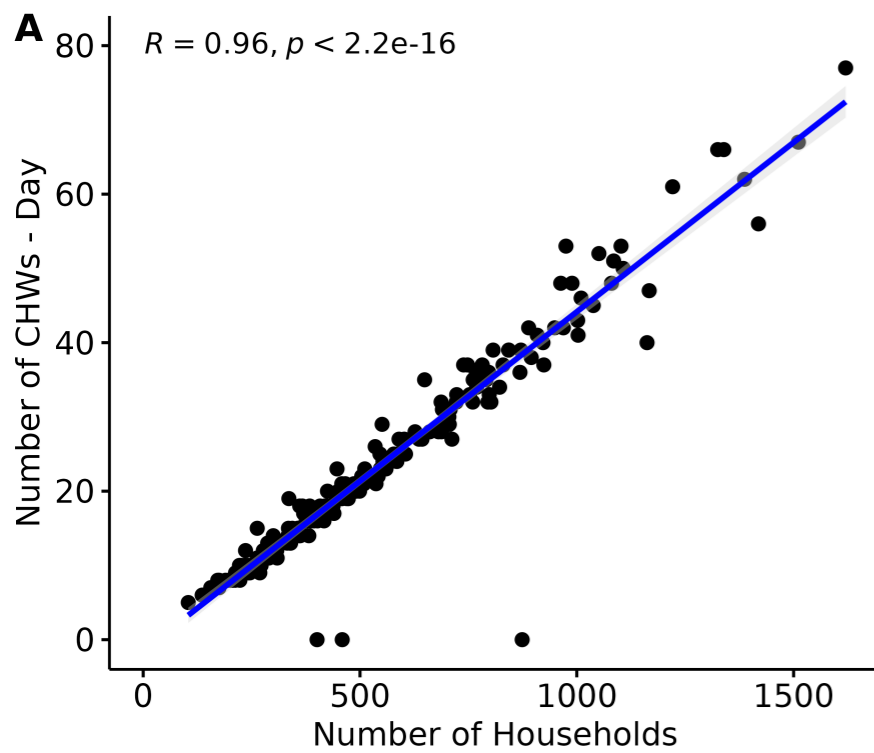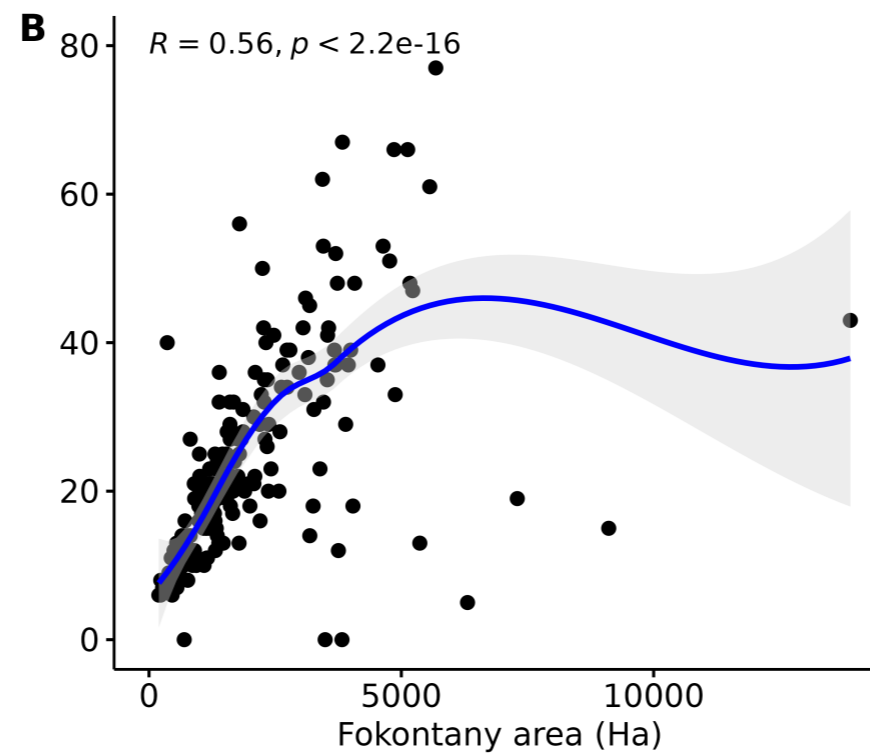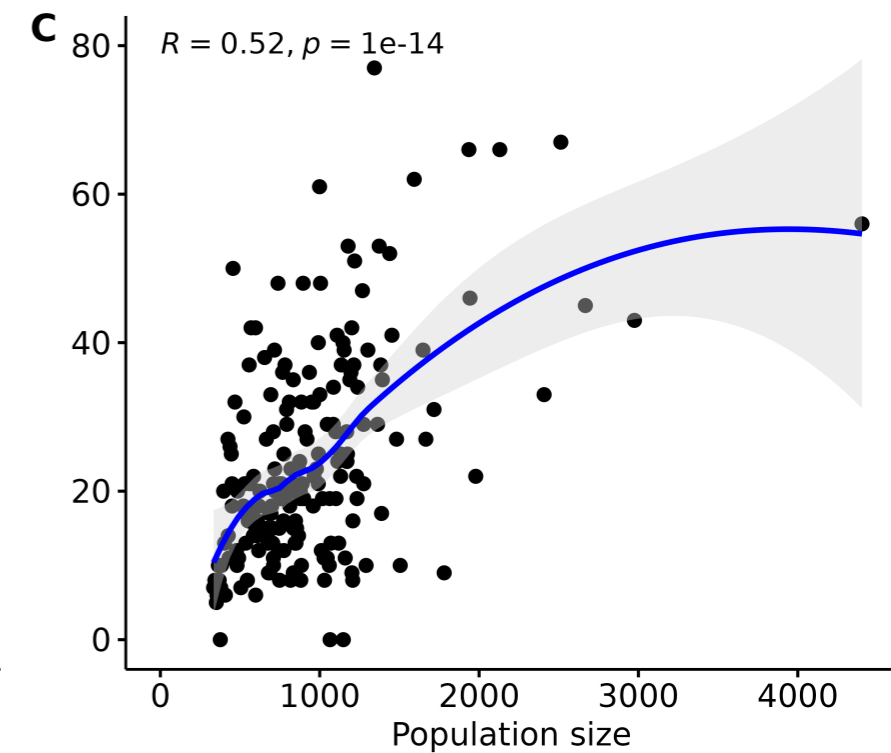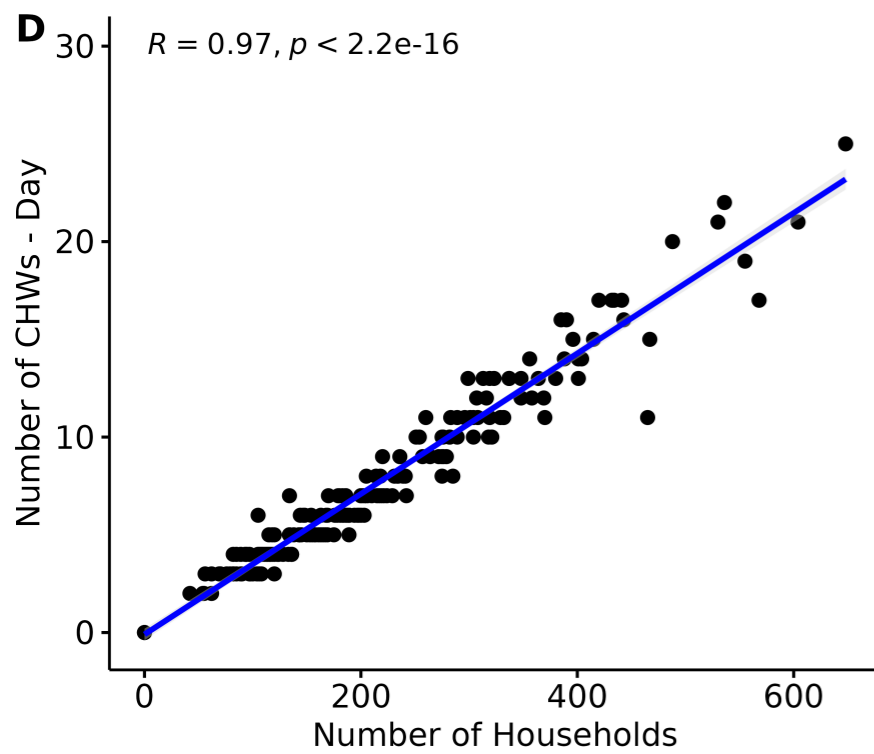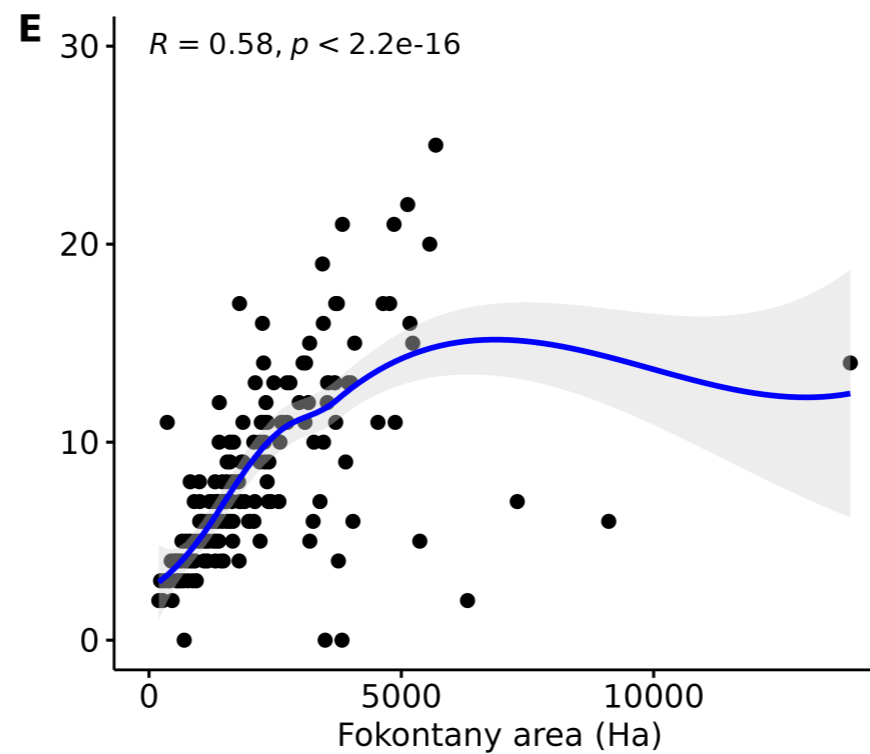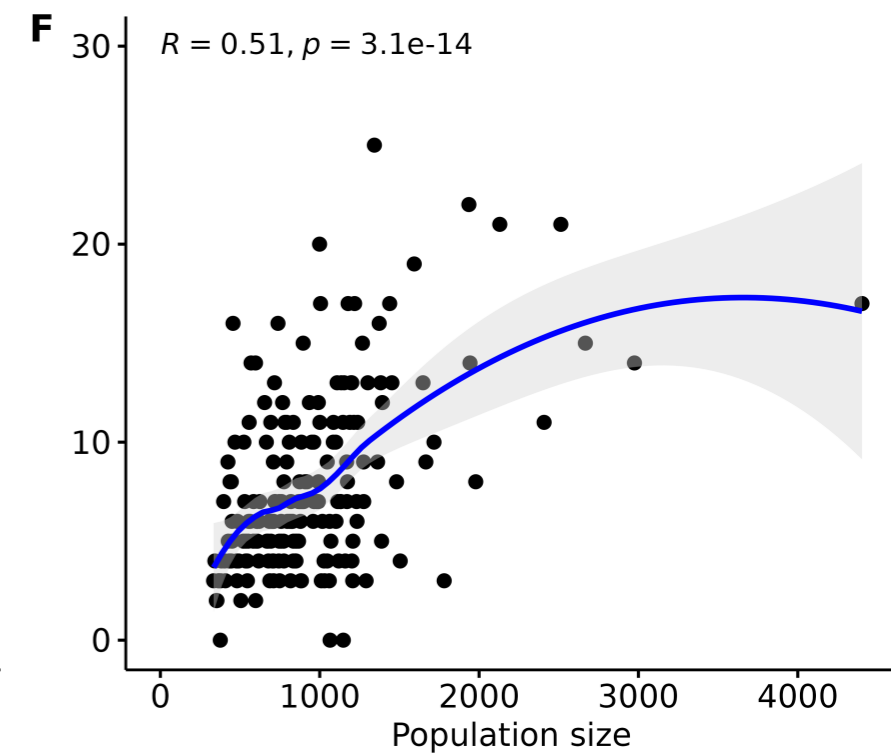

Supplement: S2 Fig — A-C: Mass administration campaigns, D-F: Proactive Community Health (one visit per month). Panels A and D plot a linear regression line due to the nature of the relationship, while the other panels use non-linear local regression fitting. (PDF) [file pdig.0000621.s002.pdf]

Ifanadiana District (Min = 1490 to Max = 1520 personnel days)

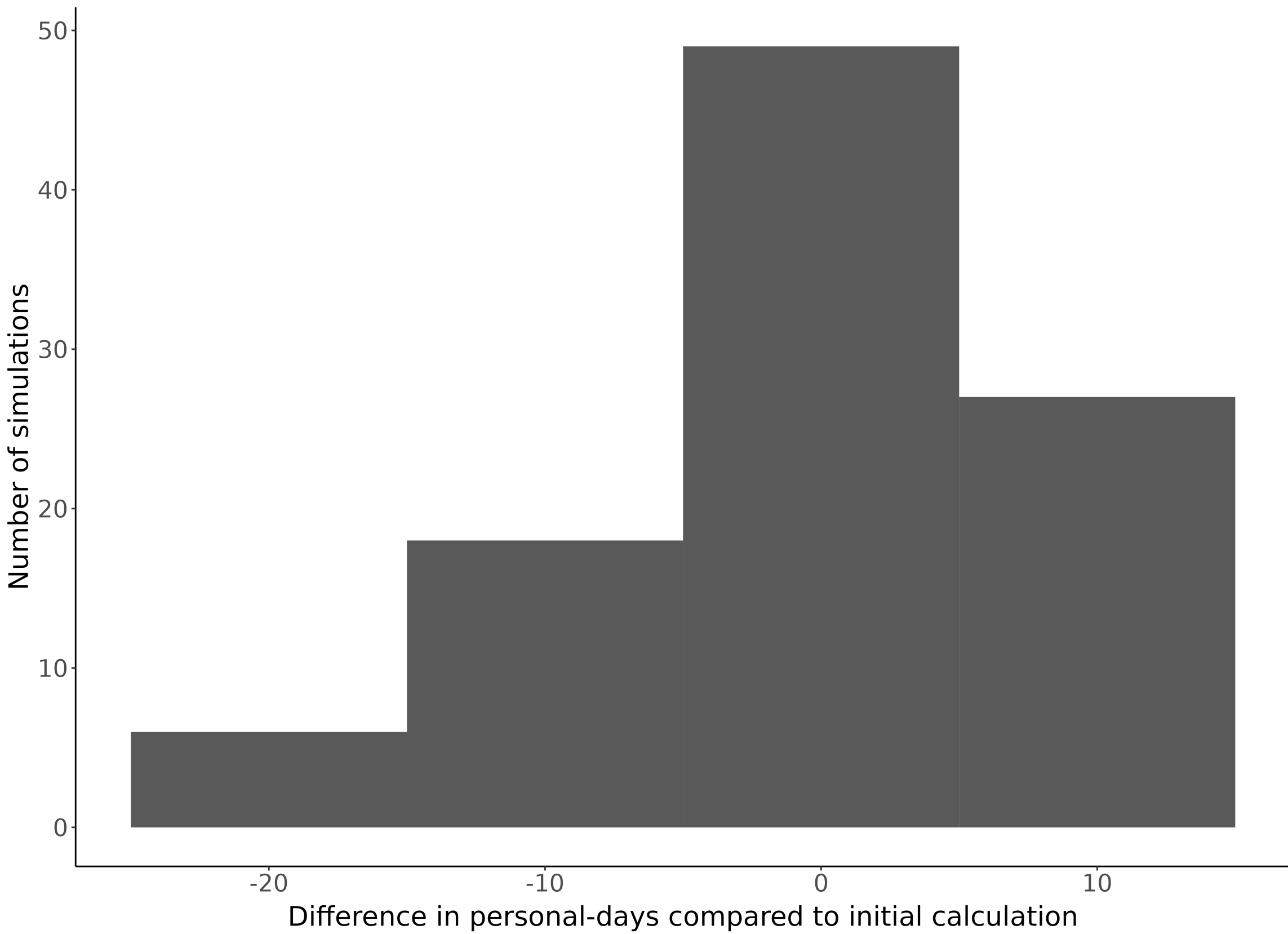

Supplement: S4 Fig — (PDF) [file pdig.0000621.s004.pdf]
