# Supplementary material for: Combining OpenStreetMap mapping and route optimization algorithms to inform the delivery of community health interventions at the last mile
Source: PLOS Digit Health. 2024 Nov 7;3(11):e0000621. doi: 10.1371/journal.pdig.0000621 (PMC11542841; doi:10.1371/journal.pdig.0000621)
Supplement: S1 Table — (PDF) [file pdig.0000621.s005.pdf]

Commune	Number of buildings	Travel distance (km)	Travel duration (h)	Personnel -Day	Personnel -Month
Ambiabe	3,583	1,767	1,222	163	11
Ambohimanga Du Sud	13,050	4,107	3,987	533	37
Ambohimiera	11,511	4,759	3,746	501	34
Ampasinambo	2,975	487	400	56	5
Analampasina	5,199	2,186	1,700	227	14
Androrangavola	8,519	3,433	2,756	368	27
Antaretra	4,636	1,524	1,430	193	15
Antsindra	5,422	2,669	1,851	245	15
Fasintsara	5,136	1,640	1,574	218	19
Ifanadiana	7,478	3,796	2,574	339	20
Kelilalina	5,347	2,000	1,697	229	18
Maroharatra	9,032	3,582	2,906	393	26
Marotoko	5,370	2,244	1,753	235	17
Ranomafana	4,182	1,797	1,372	184	13
Tsaratanana	16,488	8,505	5,706	755	48
**Total**	**107,928**	**44,496**	**34,674**	**4,639**	**319**
