# Supplementary material for: Combining OpenStreetMap mapping and route optimization algorithms to inform the delivery of community health interventions at the last mile
Source: PLOS Digit Health. 2024 Nov 7;3(11):e0000621. doi: 10.1371/journal.pdig.0000621 (PMC11542841; doi:10.1371/journal.pdig.0000621)
Supplement: S2 Table — (PDF) [file pdig.0000621.s006.pdf]

Commune	Number of households	Visit - once per month		Visit - twice per month		Visit - four times per month	
		Personnel		Personnel		Personnel	
		Day (Ref)	Month	Day	Month	Day	Month
Ambiabe	1,433	55	7	110	8	220	12
Ambohimanga Du Sud	5,221	171	23	342	27	684	45
Ambohimiera	4,604	163	21	326	27	652	41
Ampasinambo	496	19	5	38	5	76	5
Analampasina	2,081	73	9	146	11	292	18
Androrangavola	3,406	119	15	238	18	476	31
Antaretra	1,855	60	10	120	10	240	15
Antsindra	2,168	81	7	162	12	324	20
Fasintsara	2,054	77	16	154	17	308	22
Ifanadiana	2,993	112	10	224	16	448	27
Kelilalina	2,138	72	12	144	14	288	20
Maroharatra	3,616	129	19	258	23	516	34
Marotoko	2,147	76	10	152	12	304	18
Ranomafana	1,673	60	8	120	10	240	14
Tsaratanana	6,596	241	24	482	34	964	57
**Total**	**42,481**	**1,508**	**196**	**3,016**	**244**	**6,032**	**379**
