# Supplementary material for: Combining OpenStreetMap mapping and route optimization algorithms to inform the delivery of community health interventions at the last mile
Source: PLOS Digit Health. 2024 Nov 7;3(11):e0000621. doi: 10.1371/journal.pdig.0000621 (PMC11542841; doi:10.1371/journal.pdig.0000621)
Supplement: S3 Table — (PDF) [file pdig.0000621.s007.pdf]

		Difference between resampled and initial estimate		
**Initial estimate of personnel-days required**	**Number of fokontany (%)**	**Personnel – days required per fokontany**		
		**Used in manuscript (1 visit per month)**	**Minimum of 100 simulations**	**Maximum of 100 simulations**
[0,5]	74 (38.0%)	4	-1	+1
(5,10]	73 (37.4%)	8	-1	+1
(10,15]	34 (17.4%)	13	-2	+1
(15,20]	10 (5.1%)	18	-1	+1
> 20	4 (2.1%)	23	0	+1
Total district	*195 (100%)*	*1,508*	*-18*	*12*
