# Supplementary material for: Combining OpenStreetMap mapping and route optimization algorithms to inform the delivery of community health interventions at the last mile
Source: PLOS Digit Health. 2024 Nov 7;3(11):e0000621. doi: 10.1371/journal.pdig.0000621 (PMC11542841; doi:10.1371/journal.pdig.0000621)
Supplement: S1 Text — (PDF) [file pdig.0000621.s008.pdf]

Predictions obtained from the VRPTW algorithm were compared to results obtained in the field in terms of personnel-days required to understand the validity of our hypotheses and the validity of the results generated. For this, we took advantage of a previous census survey that was conducted in two communes of Ifanadiana District (Kelilalina and Tsaratanana) back in 2020 by teams from the NGO Pivot and CHWs from those communes. Since these surveys were conducted two years prior to the development of our algorithms, data collection had not been specifically recorded with the level of detail necessary for a robust validation of the tool. Instead, a group session was organized to facilitate discussions between the authors and 14 participants from the community health teams who had participated in the field surveys. The objective of this group session was to gather information regarding the human resources deployed per commune, as well as the number of days of work and the average duration of work that had been necessary for carrying out these census surveys.

The census was achieved with a team of 35 personnel working over a period of 6 days for about 11 hours per day in the Kelilalina commune, and a team of 47 personnel working over a period of 18 days for about 10 hours per day in the Tsaratanana commune. Subsequently, we standardized the calculations in terms of personnel-days to make them comparable with our own calculations (assuming a worker puts in 8 hours of work per day), which resulted in 289 personnel-days for Kelilalina and 1,058 personnel-days for Tsaratanana. To compare these observed values with the predictions obtained from the VRPTW algorithm, we used the parameters of the mass distribution campaign scenario for these two communes, which is equivalent to a full census in that all buildings in the catchment are visited and those who are inhabited households require a higher visit time.

Table. Comparison of algorithm predictions and field-observed CHW needs in Ifanadiana district

Commune	Number of buildings	Personnel-Days	
		**Observed**	**VRPTW Prediction (% difference prediction vs. observed)**
Kelilalina	5,347	289	229 (-21%)
Tsaratanana	16,488	1,058	755 (-29%)

When VRPTW predictions were compared with observed personnel-days during field surveys, the optimization algorithm predicted a 29% and 21% decrease in the number of resources necessary in Tsaratanana and Kelilalina, respectively. This result was in line with our expectations, given that CHW itineraries had not been optimized for geography during fieldwork, and highlights potential benefits of the optimization algorithm for planning and scheduling.
