# Supplementary material for: Combining OpenStreetMap mapping and route optimization algorithms to inform the delivery of community health interventions at the last mile
Source: PLOS Digit Health. 2024 Nov 7;3(11):e0000621. doi: 10.1371/journal.pdig.0000621 (PMC11542841; doi:10.1371/journal.pdig.0000621)
Supplement: Attachment — Submitted filename: Response to Reviewers.pdf [file pdig.0000621.s009.pdf]

EDITOR COMMENTS

The reviews point out several key areas that should be addressed in a revision. Futhermore, I believe the discussion could be strengthened by including some additional content relating to how these findings might be best incorporated into existing systems, or perhaps which strategies could be employed to aid in the future adoption of geographic optimization algorithms into health systems.

Dear editor,

We would like to thank you for your interest in our manuscript and the great feedback provided by the three reviewers. Below, you will find a point by point response to each of the reviewer's comments.

We have also tried to address the point made by the editor on how to best incorporate these findings into existing systems via participatory action research and implementation research in the conclusion:

"Implementation of this tool into a public health program will require a participatory approach, such as community-based participatory research or participatory action research (PAR) [52], to engage CHWs and other end-users in the refinement and deployment of the application in the field and ensure its validity among CHWs. Indeed, similar approaches have been used to identify obstacles to the adoption of mHealth tools and co-design [53] in a rural health context. The tool could also be integrated into existing mobile health tools used by CHWs, such as CommCare, to encourage its uptake. While implementation research is needed to effectively integrate these tools for real world program implementation, their scale up could help optimize community health programs in line with WHO recommendations, while taking advantage of rapid advances in quality and the quantity of data and analytics from around the world to inform the most urgent programs at the last mile."

REVIEWER #1

This article describes the methodology to inform delivery of Community health interventions through the optimization of the routing of CHW households visits. It uses a combination of OSM data and a routing algorithm to provide optimum numbers and routes for CHWs according to two types of community health interventions. The article is overall very well written with sound discussion and conclusion, and with well-made maps and Figures. It is a timely addition to the current literature on using high-resolution geospatial data to optimize the provision of health services.

We thank the reviewer for these positive comments about our manuscript and for the proposed revisions, which have helped to improve the paper.

There are several points the need to be clarified, especially in the Methods section, before the manuscript can be accepted:

- Line 156: you need to make it more explicit what you mean by unoccupied households, because it could be understood as being empty because at the time of the visit the households members are out and not available. But I understand that your assumption of 40% occupancy, given the overall population, means that 60% of the building are something else than houses else (e.g. a storage house). It would be important to state it and explain why the digitalization exercise cannot discriminate the building function.

Thank you for your comment. We agree that the way it was initially described could lead to confusion. From the manual photointerpretation of high resolution images used during the mapping

step, we are unable to determine which buildings are true households with people living in them and which are other types of buildings (administrative buildings, shops, storage, etc.). We have rewritten this part to clarify this point (lines 162-172), which now reads:

"we assumed that only 40% of the 108,000 buildings were permanently occupied households (Table 1), while the rest would be other types of buildings (e.g. administrative buildings, shops, storage spaces, secondary homes near fields, etc.)"

We also clarify why the digitalization exercise cannot discriminate the building function:

"This random allocation was used because the photointerpretation of satellite imagery used for mapping the District does not allow us to discriminate between permanently occupied households and unoccupied buildings used for other purposes. This information, which is necessary for program implementation, can be obtained via an initial census or through participatory approaches with local populations, but this was beyond the scope of our study which focused on the development of the optimization method."

- **Regarding the assumed 40% occupancy rate of the building, and the random attribution of that status to all buildings, I would have liked to see a sensitivity/uncertainty analysis on the impact of the randomization of the building attribution. Would another randomization significantly change the estimation of the number of required CHW? I suspect not, but at least it would be good to discuss it in the Discussion section, with a possible justification.**

This is a great suggestion. We had not initially done this analysis because it is resource intensive in terms of computer processing to rerun the optimization algorithm on the full district, instead opting for ensuring that the sampling of occupied buildings was indeed spatially random. However, we agree that a sensitivity analysis would further demonstrate the robustness of this method. We have therefore carried out a sensitivity analysis by performing 100 different randomisations of the occupancy state of buildings to provide an estimate of the uncertainty around these estimates. We describe this sensitivity analysis in lines 247-251:

"We also conducted sensitivity analyses on the randomization of the definition of occupied households vs. other building types for both scenarios. We created 100 new datasets of occupied household locations by randomly selecting 40% of the buildings to be permanently occupied. We then reran the optimization algorithm for the proCCM scenario, following the default model assumptions (Table 1) for each of these new datasets, and compared the number of personnel-days for each randomization with the initial estimate at both the fokontany and district level."

The results are available in the Appendix, Figure S3 and S4, Table S3. We briefly discuss these results in the results and discussion sections:

"These results were robust to the distribution of occupied households within the full building dataset, as demonstrated by a sensitivity analysis of 100 randomizations of household locations. At the fokontany scale, the greatest variation was seen in fokontany requiring between 10-15 personnel days, with a difference of up to 2 personnel-days across randomizations, although the majority of fokontany (135) experienced no difference between the initial estimate and the estimates during the resampling (S3 Fig, S3 Table). When aggregated across the district, the total number of personnel-days required ranged from 1490-1520 across the randomizations, less than a 0.02% difference from the initial estimate (S4 Fig)." (lines 301-307)

"Third, while the algorithm for proCCM as presented here, with a random allocation of buildings as inhabited or uninhabited households, can be used to estimate human resources needed during the planning phase, the actual itineraries were illustrative and not for implementation purposes. In practice, use of scheduling and itineraries for proCCM would require conducting a census of the CHW catchment in order to integrate the true location of inhabited households into the algorithm. However, a sensitivity analysis revealed that the estimates of personnel-days required were remarkably robust to the distribution of inhabited and uninhabited buildings, suggesting that human resource estimates, at least, may not require these exact locations." (lines 440-446)

- **Lines 234-237: it is great that this e-health tool is available for users. I suggest you make it explicit here that when the user changes some of the (default) simulation parameters, there is no re-run of the routing and optimization analyses, but simply a display of the pre-run set of analyses (if I understood correctly, but if analyses are ran again, you need to better explain it).**

Yes, the reviewer understood this correctly. We have now made this point explicit in the description of the e-health tool in lines 335-337:

"Due to processing limitations, these parameters were defined in advance, with ranges similar to those in Figure 3, and pre-run prior for display within the dashboard."

- **Figure 4: I was nicely surprised to see in the right panel that the routing allows for part of the route to go through neighboring fokontany. This would be worth mentioning, as some other tools might consider fokontany borders to be strict barriers, unrealistically.**

Thank you for this suggestion. We have added a sentence in the Figure 4 caption to mention this:

"Fokontany limits determine the households located in the CHW catchment, but paths outside the fokontany can be used during daily itineraries if they represent the shortest route, as illustrated in the right map."

- **Lines 398-400: you state that your hypothesis was that optimized schedules would results in lower resources needs. This lower resource needs seems to be indeed confirmed by your data in the field study presented in S1 text. However, how can you be sure that these results do not merely reflect the fact that your simulations underestimate the travel time and the duration of the visits, due to the many real-life factors that would lengthen travel and visit times? Could your data provided in S1 text be rather used to calibrate your simulation parameters?**

It is true that we cannot rule out that our method underestimates travel time. The level of reduction in resource needs seems reasonable if one assumes that some degree of optimization can be achieved, and contextual information from our field teams suggested that having the tool would have helped them reduce travel time. In the previous version of the manuscript, this limitation was explained in length in the last paragraph of the discussion (a bit disconnected from the argument made in lines 398-400). In the current version, we have merged these discussion points, as requested by the reviewer in lines 455-467. It now reads:

"In our case, the VRTPW algorithm can accurately plan interventions that are predictable and repetitive, such as a MDA program, a census, or a simple proCCM intervention (e.g. proactive detection and treatment of common childhood illnesses). However, much of a CHW's duties can involve reacting to health needs as they arise, and the factors that influence variation in their day-to-day activities are not reliably captured in ways that they can be predicted in advance. For instance, when we retrospectively compared our predictions with actual personnel deployed in the field during a census survey conducted in two communes, prior to implementation of proCCM, results

revealed that the optimization algorithm predicted a number of person-days 21%-29% lower than those deployed in each commune, in line with our hypothesis that optimized schedules would result in lower resource needs (S1 Text). However, when compared to a pilot proCCM program that has taken place in one commune since October 2019, the VRPTW algorithm predicted personnel needs substantially lower in the standard scenario (Table 3 and Figure 2B) than those actually used in the intervention. While part of this discrepancy is due to the more efficient movement of CHWs via the VRTPW optimization process, it is also due to the VRTPW's inability to capture the daily changing tasks of a CHW and other real-life factors when applied at a district-level."

We appreciate the suggestion of using field data to calibrate the parameters. However, because our field data is in aggregate (number of person hours per fokontany), we cannot use it to directly parameterize either the walking speed or length of visits. However, the walking speeds were based on field data collected as part of Ihantamalala 2020 and the average visit times were based on discussions with community health workers and supervisors. We have added additional information to this respect to the manuscript:

"While previous research in our District [19] and elsewhere [28] reveals that walking speed can vary according to local conditions in land cover, terrain, and climate, we made this choice for processing efficiency, as 5km/h was the average walking speed in Ifanadiana [19]. " (lines 157-159)

"These visitation times are in accordance with average times witnessed through Pivot's own community health program during iCCM consultations. " (lines 189-190)

In addition, we are hoping to implement a more thorough study to better understand how the tool can be applied for real program implementation using field-derived parameters in the near future.

- **Lines 417-419: are these line somehow redundant with what you say on liens 398-400 ? if so you should merge the discussion bits on the predicted lower personnel needs.**

As suggested by the reviewer, we have merged these discussion points (see above).

REVIEWER #2

The authors address a crucial issue in health service delivery: reaching the last mile, which involves providing healthcare to the most inaccessible areas. Their study focuses on a rural region in Madagascar, where they have applied an existing method in a novel context. Typically, the lack of comprehensive road networks and geographic data in low- and middle-income countries (LMICs) hinders the implementation of such models. However, continuous efforts to collect and complete this data have resulted in highly detailed spatial layers, enabling the authors to successfully run the VRPTW algorithm. The article is well-written, thorough, and has already demonstrated its utility and applicability in the field.

We thank the reviewer for these positive comments about our manuscript and for the proposed revisions, which have helped to improve the paper.

I recommend that the authors address a few minor issues to further improve the article. Feedback:

-I wonder why the authors have chosen Fokontany as catchment areas and have not defined these by travel time or distance? Is this how the local government decides on health service areas? I recommend the authors to add a justification for this decision.

Yes, the national community health policy in Madagascar is that the catchment of a community health worker is the fokontany. We have now clarified this in the text and added an appropriate reference in lines 106-112:

"In line with national community health policies, each fokontany possesses a community health site (CHS) - a physical structure where two community health workers (CHWs) provide clinical consultations such as for iCCM and malnutrition, and family planning [20] (…) while there is only one CHS per fokontany (i.e., the catchment area of CHWs), the sizes of the fokontany in the district vary by orders of magnitude"

-In addition, did the authors allow CHWs in their model to cross Fokontany borders or are the borders considered as barriers? From the map in Figure 1C it seems that some households may be closer to CHWs from the neighboring Fokontany/catchment area. Has this been included in the model? I would recommend the authors to clarify this.

Yes, the CHWs in our simulations are allowed to cross Fokontany borders, because this is closer to reality than arbitrarily preventing CHWs to cross an administrative line that does not exist in the field. The goal of Figure 1A was to illustrate the breadth of information available at the fokontany level, zooming out to the whole district in Fig 1C. We have clarified this in the caption for Figure 4 in lines 349-351, where the CHWs itineraries displayed show that a CHW can take a path outside of his/her Fokontany.

"Fokontany limits determine the households located in the CHW catchment, but paths outside the Fokontany can be used during daily itineraries if they represent the shortest route, as illustrated in the right map."

Indeed, some households are closer to community health sites in neighboring fokontany. However, national policy is that CHWs treat only those residents of their fokontany. We therefore do not allow for the visitation of households outside of each CHW's catchment.

-Line 157-159: "We randomly assigned a building's status (e.g. occupied vs. unoccupied) following the percentages above for each fokontany and ensured there was no spatial correlation in this sampling procedure. The scenarios differed primarily in the percentage of households to be visited, the frequency of visits, and the duration of each visit." Could this be cross checked with results from high resolution HRSL building footprint data or WorldPop building data?

We believe that locally sourced data is better than these global datasets that are based on artificial intelligence (HRSL) or aggregation/modelling from multiple sources of freely available data (WorldPop). From our experience in rural Madagascar, these global datasets are not very accurate when trying to apply them to our local settings. Here, we use 1) surveys done by our teams on over 1600 households in Ifanadiana District every two years to estimate household composition, 2) the latest Madagascar national census conducted by the national institute of statistics to estimate total population, and 3) buildings obtained from our mapping campaign, which was done using human mappers and standard methods that ensure systematic mapping with complete and quality information: HOT Tasking Manager software to divide the district in tiles of 1km^2, hired mappers and supervisor to conduct mapping, two-stage mapping including verification of every mapped tile by a separate user, etc. Although all this information is not explained in detail, we refer the reader in the data collection section to *Ihantamalala et al 2020,* the original study for which the mapping was done. In addition, both HRSL and WorldPop are gridded data sources, which provide population at either a 30m or 100m grid, respectively, rather than at the household level needed for the routing optimization.

-The authors choose a walking speeds of 5km/h and mention in their limitations this may be an under- or overestimation. Research in different settings has shown that travel speeds on different terrain types than tarmac roads can be lower especially considering the altitude differences in the fokonotany of interest. I would suggest the authors to further clarify their decision and to have a look at the important work done by Watmough et al (https://www.nature.com/articles/s41597-022-01274-w).

Our decision was mostly due to computer processing limitations for conducting a sensitivity analysis considering a range of walking speeds and terrains. We have done extensive work to characterize travel speed according to local terrain (land cover, terrain, climate, etc.) by calibrating a statistical model with over 1000km of walking fieldwork and then extrapolating to the shortest paths obtained from OSRM across the district (Ihantamalala et al. 2020, IJHG). The challenge in integrating this method into the VRPTW algorithm, where thousands of itineraries are estimated for each CHW and day of work, is that it requires substantial processing to extract all the associated landcover and terrain variables within the itineraries, which we did for every 100m of footpath in our previous study. This previous research indeed showed that travel speed in Ifanadiana varies according to different land covers, terrain, etc. but it also confirmed that on average local populations walk at about 5km/h, so for efficiency we decided to use this constant value. We have better explained this in the main text (Methods-Scenario assumptions) in lines 157-159 and cited the work by *Watmough et al.*:

"While previous research in our District [19] and elsewhere [28] reveals that walking speed can vary according to local conditions in land cover, terrain, and climate, we made this choice for processing efficiency, as 5km/h was the average walking speed in Ifanadiana [19]."

-Line 251-252: "To cover the 108,000 buildings in Ifanadiana district during mass distribution campaigns, a total of 4,639 person-days would be required to complete the work." Clarify whether these are inhabited buildings or all buildings.

We are assuming that in a mass distribution campaign, CHWs or health workers need to visit all buildings, whether occupied or unoccupied, and distribute the medicine/product to those where people lived. We have clarified this in lines 279-280. It now reads:

"To cover the 108,000 buildings in Ifanadiana district during mass distribution campaigns (both occupied households and unoccupied buildings), a total of 4,639 person-days would be required to complete the work."

-Line 256-258: "Fokontany with larger catchment areas and more dispersed households required a larger number of personnel compared to smaller communities with residential zones in close proximity (Figure 2A)." This sentence seems a little strange to me, because the catchment areas are the same as the boundaries of the fokontany. Suggestion to change to: "Larger fokontany with more dispersed households…"

Thank you for this suggestion, which we have integrated in the text.

-It is unclear whether the some of the building status will be fed back into the models. If CHWs visit a building that is uninhabited, will the model be updated to present a more realistic assumption rather than a random classification?

At the moment this has not been done because the tool has not yet been integrated into the existing CHW program. A thorough implementation research study needs to be done to better understand how the tool can be integrated in real program implementation of the local community health program, something that we are hoping to carry out in the near future. When the tool is used for

implementation purposes, regular updates of building occupancy will indeed be important and necessary to reflect real world conditions as close as possible.

We have clarified this in the description of the tool in lines 271-276:

"It is important to note that while the e-health tool presented here is intended for operational use, these algorithms have not yet been used by the existing CHW program in Ifanadiana District or integrated into CHWs' workflows. For this, implementation research is needed in order to tailor the general method described here to users' expectations, field constraints, particular CHW program features, or use with existing software. While this was beyond the scope of this study, such work will ensure that the tool contributes to decision making and program implementation of the local community health program."

Also in the methods section we mention in lines 167-172:

"This random allocation was used because the photointerpretation of satellite imagery used for mapping the District does not allow us to discriminate between permanently occupied households and unoccupied buildings used for other purposes. This information, which is necessary for program implementation, can be obtained via an initial census or through participatory approaches with local populations, but this was beyond the scope of our study which focused on the development of the optimization method."

REVIEWER #3

The concept behind this paper is great, and I'm glad to see an innovative route optimization approach to enhance equitable care coverage by community health workers.

We thank the reviewer for these positive comments about our manuscript and for the proposed revisions, which have helped to improve the paper.

Below are some of my comments:

Overall: Firstly, the authors need to provide more concise background information on current coverage of CHWs in the districts. There is a mention of 180 CHWs supported by NGO, but what about government CHWs, combined what does the current coverage looks like. How many people are currently not being reached? Such information is critical to justify for your innovation.

There is no separation between government and NGO CHWs: as per national policy, there are two CHWs per fokontany and all of the CHWs in the district are government CHWs (a total of 390 CHWs); about half of the CHWs in the district are supported by the NGO for training, supervision, supplies, etc.

The main challenge is that, because there is a fixed number of CHWs per fokontany and they do "passive" diagnosis and treatment at community sites, there remain barriers in access to community health for those fokontany that are larger. We have expanded the explanation of the community health program in Ifanadiana District and existing barriers to care in lines 106-123. It now reads as follows:

"In line with national community health policies, each fokontany possesses a community health site (CHS) - a physical structure where two community health workers (CHWs) provide clinical consultations such as for iCCM and malnutrition, and family planning (…).

For community health, Pivot supports the activities of 180 out of the 390 CHWs, covering half of the district. (…)

However, access to healthcare at the community level remains problematic due to geographical barriers. Less than one quarter of households live in the immediate vicinity (defined as within 1 km) of a CHS [19], and distance to the CHS is associated with a decrease in utilization by over 25% per kilometer [13]. To further reduce geographic barriers, a proCCM program was piloted in one commune, where CHWs conducted home visits to every household in their catchment at least once per month, allowing them to consult, treat children, do door-to-door sensitization and follow-up on unwell individuals within their communities [6]. "

Also, the authors didn't provide the specifics of complex CHW program (line 51-52), which is a bit vague. Although. , there is mention of If the tool is intended for program managers, as indicated on line 228, more information is needed about these users and their capacity to utilize this application. The software used in this research may not be easily transferable to community health worker supervisors or district health teams. Include a justification regarding the intended target user and its relevance.

The sentence about CHW programs becoming more complex referred mostly to the previous paragraph, where the breadth of activities they are involved in is explained in detail, but can be summarized as having increasingly larger scopes of work and target populations over time. We have added the following phrase to clarify this point in lines 53-54: *"As community health programs gain relevance within national health systems, they are becoming inherently more complex, with larger scopes of work and target populations".*

Regarding the intended users and software, we would like to clarify that although this study originated in response to observed needs and challenges regarding planning and implementation of community health delivery in our setting in rural Madagascar, the goal of this manuscript was to describe a general method that can be used in a wider range of settings and situations that involve door-to-door visits, including census activities, mass administration campaigns and some proactive community health programs. A thorough implementation research study needs to be done to better understand how the tool can be integrated in real program implementation of the local community health program, something that we are hoping to do in the near future. We clarify this in the revised version of the paper in lines 271-276:

"It is important to note that while the e-health tool presented here is intended for operational use, these algorithms have not yet been used by the existing CHW program in Ifanadiana District or integrated into CHWs' workflows. For this, implementation research is needed in order to tailor the general method described here to users' expectations, field constraints, particular CHW program features, or use with existing software. While this was beyond the scope of this study, such work will ensure that the tool contributes to decision making and program implementation of the local community health program."

Your title indicated participatory mapping, but I failed to find how this was executed. What aspect of this research involved participatory methods? If validation of routes was done by two technical people that seems more like a research team effort than community participation. For true participatory mapping, involving community members to validate digitized roads, buildings, etc., sourced from OSM, or including CHWs, is necessary. The participatory mapping aspect needs thorough and clear description.

We apologize for the misuse of the term participatory mapping. We used it as a short way to refer to the use of OSM as the main GIS data source, which is typically built thanks to the contributions of a community of volunteer mappers, mapping both locally and remotely. Although there was some local knowledge involved (feedback from our local staff on passable roads, location of health

infrastructure, etc.), this was not an integral component of our mapping activity. We agree with the reviewer that this by itself does not qualify as participatory mapping, so we have changed references to participatory mapping. For example, the title now reads

"Combining OpenStreetMap mapping and route optimization algorithms to inform the delivery of community health interventions at the last mile"

Similar changes have been made throughout the manuscript to avoid using the term participatory mapping (10 instances).

Another major flaw concerns the specifics of route optimization, particularly the random selection of uninhabited buildings. If a participatory approach was used, sampling a few buildings in-person to validate/ground truth your digitization could improve the credibility, the current approach is not sufficient as a reliable node in the optimization process. And solely relying on spatial correlation checks does not sufficiently address limitations in route selection from CHWs. The core of this paper revolves around finding the best route from CHWs to households. This is where a participatory approach could be instrumental.

We agree whole-heartedly with the reviewer. The goal of this manuscript was to describe a general method that can be used in a wider range of settings and situations that involve door-to-door visits, including census activities, mass administration campaigns and some proactive community health programs. A thorough implementation research study needs to be done to better understand how the tool can be integrated in real program implementation of the local community health program.

Regarding the specific point about the random selection, this was done in order to provide estimates for the planning phase in terms of human resource needs. For a future implementation phase of a proCCM program, either a census in each fokontany needs to be done at baseline of activities, or a participatory approach could be used involving the local population to identify the inhabited households. The example of itineraries for a CHW in Figure 3 was given for illustrative purposes. We have tried to clarified this in the text and add the potential value of participatory approaches in lines 167-172:

"This random allocation was used because the photointerpretation of satellite imagery used for mapping the District does not allow us to discriminate between permanently occupied households and unoccupied buildings used for other purposes . This information, which is necessary for program implementation, can be obtained via an initial census or through participatory approaches with local populations, but this was beyond the scope of our study which focused on the development of the optimization method."

In addition, we explain this limitation in the discussion section in lines 440-444:

"Third, while the algorithm for proCCM as presented here, with a random allocation of buildings as inhabited or uninhabited households, can be used to estimate human resources needed during the planning phase, the actual itineraries were illustrative and not for implementation purposes. In practice, use of scheduling and itineraries for proCCM would require conducting a census of the CHW catchment in order to integrate the true location of inhabited households into the algorithm."

Also, why use a vehicle route optimization approach when the primary mode of transport for CHWs is walking in hard-to-reach areas? Have you explored other route optimization or coverage mapping exercises?

At the beginning of the project we explored the different route optimization algorithms available (traveling salesman problem, vehicle routing problem, with and without resource constraints such as time windows, capacity constraints, etc.) and we decided to use the VRPTW algorithm because it was the approach that resembled the closest to the situation we were trying to optimize: 1) community health workers who always start their work from the same location (their community health site), 2) they need to visit door to door all households on uninhabited buildings in their fokontany (depending on the simulation), and 3) the need to come back to the original point of departure after a certain number of hours of work. The fact that we are modelling CHWs who walk along pathways instead of a fleet of vehicles along roads is mostly a matter of changing the travel speed and the type of pathways that they can use within the road/path network, but this approach allows us to accurately model the type of work done in the field for censuses, mass administration campaigns, and active surveillance or simple proactive community case management programs. Described simply, although vehicle routing was initially developed for vehicles, is it not limited to such, but can describe any agent moving along a transport network, such as someone walking along footpaths. We try to better explain this in the Methods, Data analysis section in lines 144-148:

"We applied the VRPTW algorithm for two scenarios, which correspond to two types of community health interventions requiring door-to-door delivery: mass distribution campaigns and proCCM programs. In both scenarios, the vehicle fleet was represented by survey teams or CHWs who walk along footpaths within a rural district (instead of driving along a road), the central node was the CHS, and the clients were the households or buildings to be visited."

In the abstract, you mention the feasibility and utility of the tool; however, the conclusion does not adequately address these intended goals.

We have made some changes so that the message in the abstract and conclusion are more in line. The abstract now reads:

"The goal of this study was to demonstrate how geographic optimization can be applied to inform community health programs in rural areas of the developing world."

The conclusion now reads:

"This study demonstrates an innovative approach to inform the planning and implementation of door-to-door delivery activities at the community level that relies on existing geographic optimization methods, by combining a Vehicle Routing Problem with Time Windows (VRPTW) algorithm with an exhaustive GIS dataset consisting of all road networks and buildings in a rural health district, obtained through OpenStreetMap mapping. The algorithm, which predicts the number of personnel-days needed to implement these activities according to program design and the characteristics of the CHW catchment, can also generate detailed schedules and itineraries for implementation by CHWs and field teams via a dedicated "e-health" platform. Implementation of this tool into a community health program will require a participatory approach, such as community-based participatory research or participatory action research (PAR) [52], to engage CHWs and other end-users in the refinement and deployment of the application in the field and ensure its validity among CHWs. Indeed, similar approaches have been used to identify obstacles to the adoption of mHealth tools and co-design [53] in a rural health context. The tool could also be integrated into existing mobile health tools used by CHWs, such as commCare, to encourage its uptake. While implementation research is needed to effectively integrate these tools for real world program implementation, their scale up could help optimize community health programs in line with WHO recommendations, while taking advantage of rapid advances in quality and the quantity of data and analytics from around the world to inform the most urgent programs at the last mile."

The background could be simplified by structuring it into introductory paragraphs:

- **What is the problem**
- **What do we know about it**
- **What do we not know about it**
- **Why did you conduct this study and why it is relevant locally or in other similar settings?**

Specifically:

Background on how the optimization tool works, even, if possible, visualize? And explain the process of adaptation for your setting.

Also, there are few places where the importance of the tool and approach is repeated in the intro, methods, results and discussion.

We have preferred to keep the current structure of the background, because we feel it covers the main issues around the importance of community health delivery, current challenges in its implementation and optimization, as well as existing tools to improve community health programs and their limitations. Information on how the optimization tool works is available in the methods, and Figure 1 attempts to describe visually and in a simple way how the tool works using data from a real setting.

We have made some changes across the manuscript to reduce repetition around the importance of the tool and approach.

Methods and results are partially merged, please discern the two sections.

We were unable to find exactly where the two sections had merged referred to by the reviewer without specific examples. However, we did notice repetition in our discussion of the e-health tool in both the Methods and Results sections. We have modified these two subsections in an attempt to make them more distinct.
